# Association between migraine and pre-eclampsia among pregnant women: a single hospital-based case–control study in India

**DOI:** 10.1186/s12884-024-06567-z

**Published:** 2024-05-16

**Authors:** Shyamashree Biswas, Ranjana Singh, A. G. Radhika

**Affiliations:** 1 Indian Institute of Public Health, Delhi, India; 2 University College of Medical Science & GTB Hospital, Delhi, India

**Keywords:** Pre-eclampsia, Migraine, India, Pregnancy

## Abstract

**Background:**

Pre-eclampsia and migraine share some similar aspects of pathophysiology such as vascular function, platelet activation, and enhanced clotting. A few observational studies from different demographics showed that pregnant women with a history of migraine were at higher risk of developing pre-eclampsia. However, there is no such evidence available from the Indian context. Hence, a hospital-based case–control study was conducted among Indian women to determine the association between migraine and pre-eclampsia.

**Method:**

It was a single-centre case-control study in a tertiary care hospital in India. Cases were pregnant women with clinically diagnosed pre-eclampsia, and controls were normotensive pregnant women. Migraine was diagnosed with a questionnaire adapted from the “International Classification of Headache Disorders (ICHD), 3rd Edition” by the International Headache Society, (IHS). We performed logistic regression to explore the association between migraine and pre-eclampsia.

**Result:**

One hundred sixty-four women (82 women per group) were enrolled. The mean age among the cases (24.5 years, standard deviation of 2.4 years) was slightly higher than the mean age of the controls (23.5 years, standard deviation of 2.5 years) with a *p*-value of 0.006. We found that women with a history of migraine were more likely to develop pre-eclampsia (Adjusted Odds Ratio 6.17; *p*-value < 0*.*001, 95% Confidence Interval of 2.85 to 13.62).

**Conclusion:**

The current findings suggest a significant association between migraine and pre-eclampsia aligning with previous study findings; nevertheless, larger follow-up studies including women from different states in India are needed.

**Supplementary Information:**

The online version contains supplementary material available at 10.1186/s12884-024-06567-z.

## Background

Pre-eclampsia is one of the leading causes of maternal and neonatal mortality and morbidity which typically affects 2–5% of pregnant women [1, 2]. According to a population-level study based on the Global Health Data Exchange (GHDx), the incidence of hypertensive disorders in pregnancy has increased from 16.30 million to 18.08 million globally, with a total increase of 10.92% from 1990 to 2019 [3]. In 2019, the number of deaths globally due to hypertensive diseases in pregnancy was approximately 27.83 thousand [3]. In low- and middle-income countries, 16% of maternal deaths are attributable to hypertensive disorders in pregnancy such as pre-eclampsia and eclampsia [4]. A systematic review including studies between 2006 and 2020, found a pooled prevalence of hypertensive disorders of pregnancy in India as 11% (95% CI, 5–17) [5].

Besides deaths, women who survive pre-eclampsia are at higher risk of developing cardiovascular diseases, diabetes, and dyslipidemia in later life [6]. Moreover, infants born from pre-eclamptic pregnancy may suffer from attention deficit hyperactivity disorder (ADHD), cardiovascular and metabolic disorders [2, 6]. Additionally, the financial burden of healthcare for pre-eclampsia management is considerable which is primarily driven by costs to treat several adverse outcomes of the newborn [7].

There are several potential risk factors identified as associated with pre-eclampsia, however, most of them lack strong power to predict the onset of pre-eclampsia [2, 8]. Chronic hypertension, chronic renal diseases, diabetes mellitus, higher body mass index (≥ 30 kg/m^2^), and systemic lupus erythematosus are some well-recognized risk factors of pre-eclampsia [2, 9].

Migraine, a very common headache disorder, may increase the risk of developing vascular diseases during pregnancy, including pre-eclampsia [10–13]. Migraine is the most common neurological disease worldwide which is primarily characterized by episodic disabling headaches with or without autonomic nervous system dysfunction [14, 15]. Migraine affects 14–15% of the global population and it accounts for 4.9% of the global burden of disease [16]. The prevalence of migraine has increased globally in the last three decades [15]. It is 2 to 3 times more prevalent among women than men affecting 20.7% of women and 9.7% of men globally [17, 18].

Though, almost 30% of women experience migraine by the age of 45 years [19], its impact on pregnancy outcome, neonatal health and later life have not been fully explored [20]. A few patients seek diagnosis and treatment from a headache specialist and others prefer to self-medicate, which makes it more difficult to prevent the consequences of long-term migraine [21].

Both pre-eclampsia and migraine have complex pathophysiology, and the linking mechanisms of these two are not fully understood [12]. Many studies reported that migraine increased the odds of developing pre-eclampsia [10–13, 19, 22, 23]. An overlapping clinical and pathophysiological features of migraine and pre-eclampsia such as inflammation, platelet activation, vascular endothelial dysfunction, and alterations in vascular reactivity may explain the association between these two [2].

A recent hospital-based case–control study of Sudan reported that pregnant women with a history of migraine were more likely to develop pre-eclampsia (AOR 9.01, 95% CI 4.81–16.86) [10]. Another large population-based cohort study in Denmark also found an increased risk of pregnancy-associated hypertension disorders, miscarriage, preterm birth and caesarean section among women with migraine [20]. A 2024 umbrella review with an updated systematic review reported that migraine was associated with an increased odds of developing pre-eclampsia (pooled OR 2.05, 95% CI 1.47- 2.84) and preterm birth (pooled OR 1.26, 95% CI 1.21–1.32) [19]. However, to our knowledge, there is no study conducted in India evaluating the association between pre-eclampsia and migraine among Indian women, and researchers reiterate that the magnitude of the association of any risk factor may vary from population to population. Furthermore, many previous studies did not adhere to the standardized criteria for migraine diagnosis. Therefore, the primary objective of our study was to assess the association between migraine and pre-eclampsia among Indian women.

## Methodology

### Study design and setting

A hospital-based case–control study of 164 participants was carried out. Participants were recruited based on their inclusion and exclusion criteria from the antenatal clinics and wards of the Department of Obstetrics and Gynaecology, Guru Teg Bahadur Hospital in Delhi. Data was collected between January to March 2022.

### Study participants

Participants were members of the source population and resided mostly in the eastern part of Delhi. All the participants were more than 18 years old, primiparous, and had no pre-pregnancy history of hypertension. Participants were screened through pre-defined inclusion and exclusion criteria. Data was collected from participants who met the inclusion criteria and gave consent for study participation. Eligibility criteria for cases and controls are mentioned below:

### Inclusion criteria

#### Cases

Cases were primiparous women, aged 18 years or more, with clinically diagnosed pre-eclampsia attending antenatal clinics or admitted to the wards. Antepartum women were interviewed any time after 20 weeks of gestational age, whereas, delivered primiparous women having pre-eclampsia were interviewed during their stay in the hospital wards.

#### Controls

Controls were postpartum, primiparous women, who had delivered the baby at term without developing pre-eclampsia on the same day or the next day of recruiting the case. Controls were interviewed at the postpartum wards once they became eligible to get discharged from the hospital.

### Exclusion criteria

Women with the following conditions were excluded from the study: being more than 35 years old, having chronic hypertension or chronic diabetes mellitus, experiencing post-partum haemorrhage, developing eclampsia with HELLP syndrome (hemolysis, elevated liver enzymes, and low platelet count), or having a history of systemic lupus erythematosus or requiring assisted ventilation for multi-organ failure. Furthermore, we excluded women who delivered stillborn babies or babies with any congenital anomalies or macrosomia.

### Operational definition

#### Pre-eclampsia

Pre-eclampsia is defined as: “A systemic syndrome characterized by a new-onset of hypertension (blood pressure systolic > 140 mm Hg, diastolic > 90 mm Hg on two occasions at least 4 h apart, or in severe cases systolic blood pressure > 160 mm Hg and diastolic blood pressure > 110 mm Hg) and proteinuria (protein/creatinine ratio of > 0*.*3 mg/dl or protein > 5 g in a 24 h urine sample, or > 3 g in two samples taken 6 h apart from a patient on bed rest) after 20 weeks of gestational age in pregnant women, which resolves before the end of 6*th* week postpartum” [24]. Pre-eclampsia may also present with hypertension along with any features of end-organ damage without proteinuria [24]. In the present study, women with clinically diagnosed pre-eclampsia were selected as cases and we collected data from hospital records.

#### Migraine

Migraine is a common chronic neurological disorder that is characterized by episodic disabling headaches with or without autonomic nervous system dysfunction [25]. It has two major types: Migraine without aura: “characterized by episodic headaches with specific features and associated symptoms”, and Migraine with aura: “headaches along with the transient focal neurological symptoms that usually precede or sometimes accompany the headache” [25]. In this study, we considered only migraine without aura because symptoms of migraine with aura may mask the signs and symptoms of severe pre-eclampsia. Migraine was diagnosed according to the International Classification of Headache Disorder (ICDH) by the International Headache Society [26]. The diagnostic criteria of migraine without aura by The International Classification of Headache Disorders, 3rd edition, are stated below [26].

#### Migraine without aura

According to the International Classification of Headache Disorder (ICDH) by the International Headache Society, migraine without aura is defined as:

- “At least five attacks fulfilling criteria B-D,
- Headache attacks lasting for 4-72 hours (when untreated or unsuccessfully treated),
- Headache has at least two of the following four characteristics: unilateral location, pulsating quality, moderate or severe pain intensity, aggravation by or causing avoidance of routine physical activity (e.g., walking or climbing stairs),
- During headache at least one of the following: nausea and/or vomiting, photophobia, and phono-phobia,
- Not better accounted for by another ICHD-3 diagnosis.”

#### Probable migraine without aura

According to the International Classification of Headache Disorder (ICDH) by the International Headache Society, probable migraine without aura is defined as:

- “Attacks fulfilling all but one of criteria A–D for migraine without aura,
- Not fulfilling ICHD criteria for any other headache disorder,
- Not better accounted for by another ICHD-3 diagnosis.”

#### Sample size and sampling

The sample size was estimated based on the primary objective of the study which was to assess the association between migraine and pre-eclampsia. The first population-based survey of India reported that the prevalence of migraine was higher among women (32.4%) than men (18.6%) [27]. Assuming the prevalence of migraine as 32.4, the odds ratio for the association between migraine and pre-eclampsia as 2.8 [28], a two-sided hypothesis with 5% alpha and a power of 90%, the sample size for this study was calculated as 82 participants in each group; a total of 164 participants. Stata 15 was used to calculate the sample size. A consecutive sampling method was used for selecting participants for the study. All the eligible participants fulfilling the inclusion criteria during the data collection period were recruited for collecting the data.

#### Study tool and data collection

A structured questionnaire was developed to collect the required information from the participants using paper forms. The database was developed in Epi Info 7.2. which was tested with dummy data before initiating the study. The data collection tool had the following sections: demographic details, pregnancy-related information, pre-eclampsia-related information, and a migraine assessment questionnaire. Data was collected from the hospital record and a face-to-face interview with each participant. Interviews were conducted in the antenatal clinic and wards of the Department of Obstetrics and Gynaecology, GTB Hospital, Delhi. Antepartum women were interviewed any time after 20 weeks of gestational age, whereas, post-partum women were interviewed during their stay in the hospital after the delivery of the baby. Maternal hospital records were reviewed to collect the antenatal history of the participant. Information on regular antenatal checkups was taken from participants during the interview. Documents of outdoor visits were assessed to confirm the regularity of antenatal checkups. Written informed consent was secured from each participant before data collection. The participants were screened for migraine using a structured questionnaire adapted from the International Classification of Headache Disorders (ICHD) by the International Headache Society, 3rd Edition [26]. The ICHD criteria for migraine diagnosis is internationally accepted for the diagnosis of primary headaches and it helps in achieving relatively high precision in the diagnosis of different types of primary headaches [29]. Since there are no available biological markers or routine neuroimaging for the diagnosis of migraine, the ICHD criteria are being applied worldwide for the diagnosis of migraine [29]. Several studies from India have used the ICHD criteria to diagnose different types of headaches including migraine [29]. The interview to diagnose migraine started with the question, “Have you ever experienced headaches?”. If the response was positive, the remaining questions were asked. The participants were also asked about the frequency and duration of headache episodes, age of onset of headache, occurrence of headache episodes in the last year, and whether they availed any treatment for headache. The questionnaire was used to collect the information and after getting the responses to all the questions, the criteria of the International Headache Society for migraine diagnosis were applied to diagnose migraine. The questionnaire was developed in English after consulting a neurologist. It was translated into the local language (Hindi); further, it was back-translated into English by a third person. A pilot testing of the questions was performed during the first week of data collection to check the level of understanding of the participants and the time taken for interviewing the participants. Participants in the pilot testing were included in the final analysis as there was no modification in the questionnaire. Collected data in the paper forms were entered in the Epi Info 7.2 database. The English version of the questionnaire is available as Supplementary File.

Names or any personal identifiers of the participants were not collected. Participants were given unique serial numbers during data collection. The database was password protected and access to data was kept restricted only to those who were closely involved in data analysis. Necessary approvals from the independent ethics committee of the Indian Institute of Public Health, Delhi, and Guru Teg Bahadur Hospital were secured before initiating the study.

### Statistical analysis

The collected data was imported into Stata 15.1 for cleaning and analysis. Descriptive statistics were used to describe the characteristics of cases and controls included in the study. Mean ± standard deviation for continuous variables and frequency along with percentages for categorical variables have been reported. Independent t-test and Chi-square test were performed for the continuous and categorical variables respectively to compare characteristics between cases and controls.

The outcome of interest was pre-eclampsia, key exposure was a history of migraine, and a priori confounders were selected based on previous evidence which included age, body mass index (BMI), educational status of the mother, occupation during pregnancy, anaemia during pregnancy, and smoking status of the husband. The participants diagnosed with migraine were further classified as strict migraine without aura (fulfilling all the criteria of ICHD-3rd Edition, Code 1.1); and probable migraine without aura (ICHD-3rd Edition, Code 1.5.1). Furthermore, the women with migraine were categorized as severe, moderate, and mild migraine based on their capacity for routine activity (physical/intellectual) during headache attacks [30].

The association between migraine and pre-eclampsia was assessed by performing binary logistic regression with a significance level set at 0.05. Initial uni-variable analyses, with key exposure (migraine) as well as other exposure variables, were carried out to determine the unadjusted odds ratios (ORs) and 95% confidence intervals (CIs). All the a priori confounders from the literature were added to the final multivariable model irrespective of their significance level in uni-variable analyses. In addition to this, a few variables which were significant in the uni-variable analysis were also included. As 95% of women were housewives during pregnancy, we excluded occupation from the multivariable analysis. The final regression model included age, BMI, education status, anaemia during pregnancy, smoking status of the husband, and family history of diabetes mellitus as confounders. The fit of the final model was tested by Hosmer and Lemeshow’s goodness of fit test. We reported the results in a tabular form as odds ratio along with 95% CI, and *p*-value.

## Result

### Socio-demographic characteristics

A total of 164 participants were included in the analysis. The demographics and family history of cases and controls are compared and summarized in Table 1. The mean age among the cases was 24.5 years (standard deviation of 2.4 years), slightly higher than the mean age of the controls 23.5 years (standard deviation of 2.5 years).

**Table 1:** Distribution and comparison of demographics and family history between cases and controls

Factor	Controls n (%)	Cases n (%)	*p*^1^
***N* = 164**	***n* = 82**	***n* = 82**	
Age, mean (sd)	23.5 (2.5)	24.5 (2.4)	0.006
Body mass index (BMI)^2^	52 (63.0)	34 (41.0)	0.005
Below normal/Normal Overweight/obese	30 (37.0)	48 (59.0)	
Education ^3^			0.628
Till primary schooling	29 (35.0)	32 (39.0)	
Secondary schooling/graduation	53 (65.0)	50 (61.0)	
Monthly household income, less than 60000 rupees	75 (91.0)	73 (89.0)	0.790
Food habit			0.420
Vegetarian	27 (33.0)	32 (39.0)	
Non-vegetarian	55 (67.0)	50 (61.0)	
Religion			0.510
Hindu	51 (62.0)	56 (68.0)	
Muslim	31 (38.0)	26 (32.0)	
Occupation, *n* = 162^4^			0.810
At home doing household chores	77 (95.0)	78 (96.0)	
Others	4 (5.0)	3 (4.0)	
Smoking habit of husband			0.529
Smoker	38 (46.0)	34 (41.0)	
Non-smoker	44 (54.0)	48 (59.0)	
Family history of diabetes	5 (6.0)	16 (20.0)	0.018
Family history of hypertension	11 (13.0)	19 (23.0)	0.110

^1^p for age is from a t-test and others are from a chi-square test

^2^*BMI* Body Mass Index; In the below normal/normal BMI category, the majority had a normal BMI, and in the overweight/obese category, the majority were overweight, very few were obese

^3^In the primary schooling category, most women had completed primary education, and only a few did not have formal education. In the second category (secondary/graduation, most women had their secondary education)

^4^Missing information for two participants

Most of the participants (61% of cases and 65% of controls) had secondary schooling or graduation. Majority of the participants were housewives. About 23% of cases and 13% of controls reported a family history of hypertension. About 59% of cases and 54% of the controls reported their husbands as non-smokers and 20% of cases and 6% of controls reported the family history of diabetes (Table 1).

### Pregnancy-related characteristics

Pregnancy-related information of cases and controls are compared and summarized in Table 2. 91% of the cases and controls had regular ante-natal check-ups. 39% women among the cases and 10% among the controls took medications other than iron-calcium supplements during pregnancy. However, we did not collect details of the medications.

**Table 2:** Distribution and comparison of pregnancy-related history between cases and controls

Factor	Controls n (%)	Cases n (%)	*p*
***N* = 164**	***n* = 82**	***n* = 82**	
Regular antepartum check-ups	75 (91.0)	74 (91.0)	1.000
Anaemia during pregnancy	19 (23.0)	21 (26.0)	0.716
Took iron-calcium supplements	71 (88.0)	73 (89.0)	0.810
Took any other medication	8 (10.0)	32 (39.0)	< 0.001
Used oral contraceptives before current pregnancy	2 (2.0)	6 (7.0)	0.280

### Migraine and related details

**Fig. 1:**
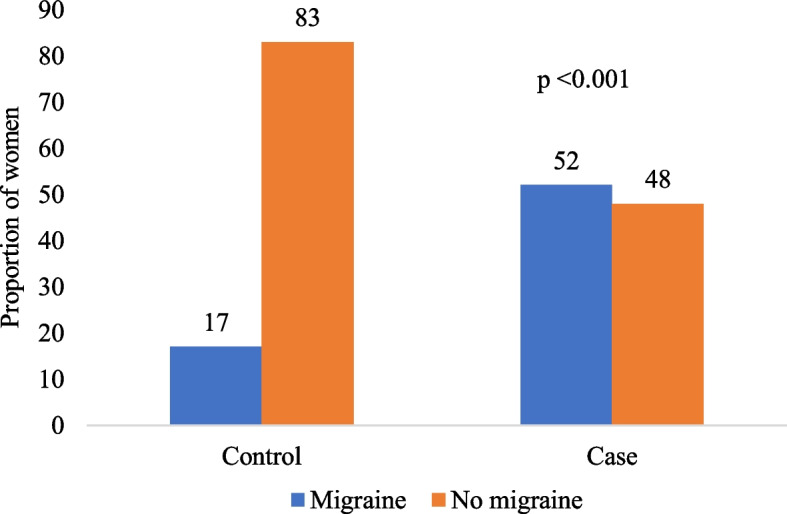
Comparison of the history of migraine according to the ICHD criteria between cases and controls

Among 164 participants, 139 participants (84.8%) reported having headaches at least once in their lifetime (Table 3). After applying the International Headache Society criteria of migraine diagnosis (ICHD Code 1.1), 43 (52%) cases and 14 (17%) controls were diagnosed with migraine without aura (Fig. 1). The comparison between the two groups demonstrated a significant difference in their history of migraine (chi-square *p*-value < 0*.*001, Fig. 1).

**Table 3:** Distribution and comparison of the history of headache between cases and controls

Factor *N* = 164	Level	Total	Controls n (%) *n* = 82	Cases n (%) *n* = 82	*p*
Ever had headache	Yes	139 (84.8)	66 (80.5)	73 (89.0)	0.128
	No	25 (15.2)	16 (19.5)	9 (11.0)

Additional information about migraine episodes among women with migraine is presented in Table 4. The participants were categorized as probable migraine and strict migraine based on the ICHD criteria. 85% of women among cases and 71% among controls were diagnosed as strict migraine. Further, participants with migraine were categorized as severe, moderate, and mild migraine based on their ability to perform regular physical activity or household work while having a migraine episode. Most of the participants with migraine had characteristics of mild attacks (72.5% cases vs. 69.2% controls). Majority of the participants with migraine reported experiencing at least one headache episode during their pregnancy (80.0% of cases vs. 85.7% of controls). Most of the cases and controls were not diagnosed with migraine earlier by a clinician (78.6% controls vs. 75.0% cases).

**Table 4:** Additional information about characteristics of migraine headaches among cases and controls

Factor *n* = 54^a^	Total	Controls n (%) *n* = 14	Cases n (%) *n* = 40
Type of migraine (*n* = 53)			
Strict	43 (81.0)	10 (71.0)	33 (85.0)
Probable	10 (19.0)	4 (29.0)	6 (15.0)
Severity^b^ (*n* = 53)			
Mild	38 (71.7)	9 (69.2)	29 (72.5)
Moderate	12 (22.6)	4 (30.8)	8 (20.0)
Severe	3 (5.7)	0 (0)	3 (7.5)
Had any headache episodes during pregnancy	44 (81.5)	12 (85.7)	32 (80.0)
Headache episodes once in every month	31 (57.4)	7 (50.0)	24 (60.0)
Migraine diagnosed by a doctor	13 (24.0)	3 (21.4)	10 (25.0)
Had medicine for headache	27 (50.0)	5 (35.7)	22 (55.0)
Hospitalized for headache at least once	3 (5.6)	1 (7.1)	2 (5.0)

^a^Though total of 57 women were diagnosed with migraine, the additional information is available for 54 women

^b^Based on their ability to perform regular physical activity or household work while having a migraine episode

### Association between migraine and pre-eclampsia

We assessed the association between migraine and pre-eclampsia using multivariable logistic regression after adjusting for relevant confounders and covariates. We also performed uni-variable logistic regression with migraine and other exposures to explore the individual effect of those exposures on pre-eclampsia.

In the uni-variable analysis, we found that the odds of having a history of migraine was 5.35 (95% CI 2.60, 11.0; *p*-value < 0*.*001) times higher among women with pre-eclampsia compared with normotensive women (Table 5).

**Table 5:** Association of migraine and pre-eclampsia, unadjusted and adjusted estimates

**Exposure variables**	**Level**	**Controls n (%) *n* = 82**	**Cases n (%) *n* = 82**	**Unadjusted OR 95% CI, p**	Adjusted OR^*^ **95% CI, p**
Migraine	No migraine	68 (83.0)	39 (48.0)	Reference	Reference
	Migraine	14 (17.0)	43 (52.0)	5.35 (2.60, 11.00) < 0.001	6.17 (2.85, 13.62) < 0.001
Age, mean (sd^a^)		23.5 (2.5)	24.5 (2.4)	1.20 (1.05, 1.38) 0.008	1.20 (1.03, 1.39) 0.018
BMI^b^	Below normal or normal	52 (63.0)	34 (41.0)	Reference	Reference
	Overweight/obese	30 (37.0)	48 (59.0)	2.44 (1.30, 4.58) 0.005	1.81 (0.94, 3.74) 0.104
Education	Till primary	29 (35.0)	32 (39.0)	Reference	Reference
	Secondary Or Graduation	53 (65.0)	50 (61.0)	0.85 (0.45, 1.61) 0.628	0.52 (0.23, 1.13) 0.099
Anaemia	No anemia	63 (77.0)	61 (74.0)	Reference	Reference
	Anemia	19 (23.0)	21 (26.0)	1.14 (0.55, 2.23) 0.716	0.69 (0.29, 1.61) 0.385
Smoking habit of husband	No	44 (54.0)	48 (59.0)	Reference	Reference
	Yes	38 (46.0)	34 (41.0)	0.82 (0.44, 1.52) 0.529	0.76 (0.36, 1.55) 0.447
Family history of DM^c^	No	77 (94.0)	66 (80.0)	Reference	Reference
	Yes	5 (6.0)	16 (20.0)	3.73 (1.29, 10.73) 0.015	3.24 (0.98, 10.69) 0.054

^*^ adjusted for age, BMI, education, anaemia, smoking habit of the husband, and family history of diabetes mellitus

^a^*SD* Standard deviation

^b^*BMI* Body mass index

^c^*DM* Diabetes mellitus

Among other exposure variables, age (OR 1.20, 95% CI 1.05, 1.38, p-value 0.008); BMI during pregnancy (OR 2.44, 95% CI 1.30, 4.58, p-value 0.005); family history of Diabetes Mellitus (OR 3.73, 95% CI 1.29,10.73, *p*-value 0.015) were significant factors associated with pre-eclampsia (Table 5).

We adjusted the final multivariable model for age, BMI, education, anaemia, smoking habit of the husband, and family history of diabetes mellitus to see the association between pre-eclampsia and migraine. We found that the odds of having a history of migraine was 6.17 (95% CI 2.85,13.62, *p*-value < 0*.*001) times higher among women with pre-eclampsia when compared to normotensive pregnant women.

## Discussion

Migraine significantly affects day-to-day functioning and impacts the overall quality of life. Despite evidence of being more prevalent among young women, its consequences on pregnancy remain inadequately studied. The current case–control study indicated a significant association between migraine and pre-eclampsia among pregnant women. To the best of our knowledge, this was the first observational study conducted to explore the association between migraine and pre-eclampsia among Indian women.

The age-standardized one-year prevalence of migraine among women was reported as 32.4% in a population-based survey conducted in Karnataka, India [27]. Another survey in Eastern India observed about 14.2% of people had migraines and the majority of them were women aged between 30–34 years [29]. A recent review about the public health importance of headaches in India reported that approximately one-fourth to one-eighth of Indian individuals had migraine [31].

In the current study, among 164 participants, 34.8% (57 women) were diagnosed with migraine without aura after applying the International Headache Society criteria of migraine diagnosis (ICHD code 1.1). A retrospective study from the Bronx, NY demonstrated that a significant proportion of women with migraine developed pre-eclampsia during pregnancy, and most of them had migraine without aura [32]. We included women experiencing migraine without aura in this present study. The decision was made to simplify the diagnosis, as the symptoms of aura could mask the symptoms of severe eclampsia. Additionally, recent studies observed that the risk of developing pre-eclampsia was increased with migraine regardless of the presence of aura symptoms [12].

The current findings indicated that age had an independent effect on the occurrence of pre-eclampsia (OR 1.20, 95% CI 1.05, 1.38, p-value 0.008), and previous studies reported the similar findings [33]. According to the findings of the current study, only a few women with migraine had been diagnosed by a clinician before the study. It potentially indicated the ignorance among women and their family members regarding women’s health and well-being, as well as their unawareness about the consequences of migraine.

In a large retrospective cohort study, it was reported that overweight and obese women had an increased risk of developing pre-eclampsia [34]. In the present study, we also observed that women with higher BMI during pregnancy were at increased risk of developing pre-eclampsia (OR 2.44, 95% CI 1.30, 4.58, p-value 0.005,). However, we did not collect information about pre-pregnancy BMI in our study.

A case–control study conducted in Washington revealed that both maternal and paternal history of diabetes were associated with an increased risk of pre-eclampsia [35]. In the present study, we also observed a significant association between a family history of diabetes mellitus and pre-eclampsia (OR 3.73, 95% CI 1.29,10.73, p-value 0.015).

The primary aim of the current study was to assess the association between migraine and pre-eclampsia and we observed a significant association between migraine and pre-eclampsia. We found that women having a history of migraine headaches are at higher risk of developing pre-eclampsia during pregnancy (adjusted OR 6.17; 95% CI 2.85 to 13.62; *p*-value < 0*.*001). The wider confidence interval might be explained due to very small number of women with migraine especially in the control group. Some unaccounted confounders in the final model might also influence the wider confidence interval.

The findings of the present study were aligned with those of recent studies [19, 20, 22]. A recent study estimating associations of self-reported migraine with adverse obstetric outcomes reported that women with a pre-pregnancy history of migraine were at higher risk of preterm delivery, gestational hypertension, and pre-eclampsia compared with no migraine [33]. Another case–control study conducted in Africa reported a significant association between a history of migraine and pre-eclampsia [10].

Though the findings were significant, the present case–control study had a set of limitations. Firstly, due to their inherent nature, these findings lacked certainty about the causal relation between migraine and pre-eclampsia. Secondly, though the findings were adjusted with few known confounders based on previous literature, there might be other known and unknown factors confounding this association. Existing findings reported that pre-pregnancy medication history had an influence on the development of pre-eclampsia [2], however, due to lack of feasibility we could not collect detailed information about pre-pregnancy history. Thirdly, as women with pre-eclampsia might have also experienced headaches, there could be overlapping symptoms which could result in misdiagnosis of migraine.

Fourthly, most of the information in this study was self-reported by the patients. In some cases, the patient might have over-reported their characteristics of headache; while others might have under-reported them because of hesitancy. So, self-reporting might have introduced reporting bias. Additionally, as we asked them about their pre-pregnancy history, so there was a chance of recall bias.

Finally, we conducted a single hospital-based study due to constraints in resources and feasibility. As a result, our findings should be interpreted with caution when attempting to generalize to a broader population.

Besides limitations, the study contained several strengths as well. To the best of our knowledge, this was the first study exploring the association between migraine and pre-eclampsia in the Indian context. All the patients’ information sheets and questionnaires for interviews were provided in Hindi and in English ensuring comprehension for study participants. Additionally, a pilot testing of the questionnaire was performed during the first week of data collection to gauge participants’ understanding. The cases (women with pre-eclampsia) were clinically diagnosed by the attending clinician, while migraine was diagnosed with the standard definition by the International Headache Society and data was collected through a questionnaire developed after consulting a neurologist. Moreover, the findings gained from the present study will contribute to the existing pool of evidence concerning the association between migraine and pre-eclampsia.

## Conclusion and recommendation

Improving maternal health is the most crucial to fight against poverty and inequality. It is well established that migraine is more prevalent among women during their reproductive age, however, the consequences of migraine during pregnancy are understudied. Moreover, migraine often remains unrecognized due to a lack of awareness. Further investigations for specific antenatal interventions among women with migraine, could potentially safeguard both mother and child, thereby contributing to achieving the Sustainable Development Goals in the long run. Although we are aware of the inherent biases of case–control studies, the current study suggested the existence of an association between migraine and pre-eclampsia. Future larger follow-up studies including women from different stated of India are warranted to explore their causal relationship.

### Supplementary Information

Supplementary Material 1.

## Data Availability

The datasets used and/or analyzed during the present study are available from the corresponding author upon reasonable request.
