# Supplementary material for: Association between migraine and pre-eclampsia among pregnant women: a single hospital-based case–control study in India
Source: BMC Pregnancy Childbirth. 2024 May 16;24:373. doi: 10.1186/s12884-024-06567-z (PMC11100195; doi:10.1186/s12884-024-06567-z)
Supplement: Supplementary file 1 — Supplementary Material 1. [file 12884_2024_6567_MOESM1_ESM.pdf]

Association between migraine and pre-eclampsia among pregnant women: a single hospital-based case-control study in India

QUESTIONNAIRE

Patient id:

Date of interview:

Participation: *Women with Pre-eclampsia (the case group) and women without it (the control group), decision by the clinician*

a. ☐ Case

b. ☐ Control

Demographic details

1) Age of the participant (in years) _________
2) Height of the participant (in cm) __________
3) Current weight of the participant (in kg) _________
4) Weight before pregnancy (in kg) __________
5) Weight gained after getting pregnant (in kg) _________
6) Literacy:

   a) ☐ 0. Literate without formal education

   b) ☐ 1. Primary education (1-4yrs)

   c) ☐ 2. Secondary(>4yrs)

   d) ☐ 3. Graduation/others

7) Diet:

   a) ☐ 0. Veg

   b) ☐ 1. non-veg

8) Annual household income (rupees)

   a) ☐ 0. Less than 60,000

   b) ☐ 1. 60,000 to 1,20000

   c) ☐ 2. More 1,20,000

9) Religion

   a) ☐ 0. Hindu

   b) ☐ 1. Muslim

   c) ☐ 2. Sikh

   d) ☐ 3. Christian

   e) ☐ 4. Others

10) Occupation during pregnancy

   a) ☐ 0. At home doing housework

   b) ☐ 1. Skilled Manual job

   c) ☐ 2. Unskilled Manual job

   d) ☐ 3. Student

e) ☐ 4. Business

11) Age of Menarche (in years) _______

12) Frequency of menstruation

a) ☐ 0. Irregular

b) ☐ 1. Regular

13) Use of Oral contraceptives before pregnancy:

a) ☐ 0. No

b) ☐ 1. Yes

Pregnancy-related information

14) Gestational week _______

15) Antenatal Check-ups

a) ☐ 0. No ANC

b) ☐ 1. Regular ANC visit

16) Iron/Calcium supplementation during pregnancy

a) ☐ 0. No

b) ☐ 1. Yes

17) Any other medication

a) ☐ 0. No

b) ☐ 1. Yes

18) Comorbidities

a) ☐ 0. Anaemia

b) ☐ 1. Urinary tract infection

c) ☐ 2. Others

19) Reason for hospitalization (text) ______

20) Maternal Hb in the latest report? _______

21) Family history of hypertension

a) ☐ 0. No

b) ☐ 1. Yes

22) Family history of Diabetes

a) ☐ 0. No

b) ☐ 1. Yes

23) Smoking status of husband

a) ☐ 0. No

b) ☐ 1. Yes

Migraine assessment

Specific Questionnaire to diagnose Migraine based on the International Classification of headache disorder, (3rd Edition).

24) Have you ever experienced a headache?

a) ☐ 0. No

b) ☐ 1. Yes

If the response for 1 is negative, the participant does not need to be interviewed further

25) How many times have you faced headaches in your life?

a) ☐ 0. Only once

b) ☐ 1. 2-4 times

c) ☐ 2. More than 5 times

26) Do your headaches usually last for more than 4 hours?

a) ☐ 0. No

b) ☐ 1. Yes

27) How long does your headache usually last if you do not take any headache medicine or the medicine is not effective?

a) ☐ 0. less than 4 hours

b) ☐ 1. 4hrs-one day

c) ☐ 2. 2-3 days

d) ☐ 3. More than 3 days

28) Is your headache one-sided?

a) ☐ 0. No

b) ☐ 1. Yes

29) How do you describe your headache?

a) ☐ 0. Not pulsating/ Not throbbing

b) ☐ 1. Pulsating/ Throbbing

30) Does the headache get aggravated by routine physical activity (as walking or climbing stairs)?

a) ☐ 0.No

b) ☐ 1. Yes

31) Does headache limit your daily functioning?

a) ☐ 0. No

b) ☐ 1. Yes

32) Do you feel nauseous when you have a headache?

a) ☐ 0.No

b) ☐ 1. Yes

33) Do you vomit when you have a headache?

a) ☐ 0. No

b) ☐ 1. Yes

34) Does the noise bother you when you have a headache?

a) ☐ 0. No

b) ☐ 1. Yes

35) Does light bother you when you have a headache?

a) ☐ 0.No

b) ☐ 1. Yes

36) Diagnosis of Migraine as per the International Classification of headache disorder, 3rd Edition. *(Based on the responses from question no 1 to 12)*

a) ☐ 0.No

b) ☐ 1. Yes

Migraine related history:

37) Age of onset of headache? (In years) ______

38) Did you get a headache last year?

a. ☐ 0. No

b. ☐ 1. Yes

39) How many times have you had a headache in the last year?

c. ☐ 0. Cannot remember of headache episode last year

d. ☐ 1. 2-3 times

e. ☐ 2. 3-5 times

f. ☐ 3. more than 5 times

40) Do you get headaches every month?

g. ☐ 0. No

h. ☐ 1. Yes

41) How many days in a month does the headache?

i. ☐ 0. Less than 5 days

j. ☐ 1. 5-14 days

k. ☐ 2. More than 15 days

42) Did you get a headache during pregnancy?

l. ☐ 0. No

m. ☐ 1. Yes

43) Have you ever been diagnosed with a migraine by a doctor?

n. ☐ 0. No

o. ☐ 1. Yes

44) Have you ever had any imaging for your headache?

p. ☐ 0. No

q. ☐ 1. Yes

45) Have you ever taken headache medicine from the doctor?

r. ☐ 0. No

s. ☐ 1. Yes

46) Have you ever been hospitalized for a headache attack?

t. ☐ 0. No

u. ☐ 1. Yes

47) How would you define your headache?

v. ☐ 0. Mild; Not so high as to limit your daily activities

w. ☐ 1. Medium; High enough to limit, but not prevent, your daily activities

x. ☐ 2. Severe: so high that it causes and compels you to avoid daily activities

48) Type of migraine as per the International Classification of headache disorder, 3rd Edition

y. ☐ 0. Probable migraine without aura

z. ☐ 1. Strict migraine without aura
